# ROSIE: RObust Sparse ensemble for outlIEr detection and gene selection in cancer omics data

**DOI:** 10.1177/09622802211072456

**Published:** 2022-01-24

**Authors:** Antje Jensch, Marta B. Lopes, Susana Vinga, Nicole Radde

**Affiliations:** 1 Institute for Systems Theory and Automatic Control, 9149 University of Stuttgart, Germany; 2 Center for Mathematics and Applications (CMA), NOVA School of Science and Technology, Caparica, Portugal; 3 NOVA Laboratory for Computer Science and Informatics (NOVA LINCS), NOVA School of Science and Technology, Caparica, Portugal; 4 INESC-ID, Instituto Superior Técnico, 72971 Universidade de Lisboa, Portugal; 5 IDMEC, Instituto Superior Técnico, Universidade de Lisboa, Portugal

**Keywords:** Ensemble, classification, robust, sparse, outlier, biomarker, triple-Negative Breast Cancer, feature selection

## Abstract

The extraction of novel information from omics data is a challenging task, in particular, since the number of features (e.g. genes) often far exceeds the number of samples. In such a setting, conventional parameter estimation leads to ill-posed optimization problems, and regularization may be required. In addition, outliers can largely impact classification accuracy.

Here we introduce ROSIE, an ensemble classification approach, which combines three sparse and robust classification methods for outlier detection and feature selection and further performs a bootstrap-based validity check. Outliers of ROSIE are determined by the rank product test using outlier rankings of all three methods, and important features are selected as features commonly selected by all methods.

We apply ROSIE to RNA-Seq data from The Cancer Genome Atlas (TCGA) to classify observations into Triple-Negative Breast Cancer (TNBC) and non-TNBC tissue samples. The pre-processed dataset consists of 16,600 genes and more than 1,000 samples. We demonstrate that ROSIE selects important features and outliers in a robust way. Identified outliers are concordant with the distribution of the commonly selected genes by the three methods, and results are in line with other independent studies. Furthermore, we discuss the association of some of the selected genes with the TNBC subtype in other investigations. In summary, ROSIE constitutes a robust and sparse procedure to identify outliers and important genes through binary classification. Our approach is ad hoc applicable to other datasets, fulfilling the overall goal of simultaneously identifying outliers and candidate disease biomarkers to the targeted in therapy research and personalized medicine frameworks.

## Introduction

Genomics, proteomics, metabolomics, transcriptomics - omics data exist in a wide variety and enable research in just as many medical fields. For example, omics data have been applied in the fields of toxicology (e.g., Thomas et al. ^1^, Sutherland et al. ^2^), nutritional science (e.g., Zhang et al. ^3^, Kato et al. ^4^) and disease research (e.g., Kan et al. ^5^, Reid et al. ^6^, Anda-Juregui and Hernndez-Lemus ^7^, Paczkowska et al. ^8^). The extraction of novel information from omics data is challenging. In particular, classification based on transcriptomics data is hampered by a large feature space and a comparably low number of individuals ( n≪p ), leading to ill-posed optimization problems. The large p , small n setting is one important problem of the curse of dimensionality and requires a special treatment. A variety of sparse methods that reduce the dimensionality of the feature space have been proposed in this context. Examples include data-based statistical methods such as Linear Discriminant Analysis ^9,10^, penalized likelihood functions ^11^, variable selection methods or shrinkage approaches ^12^, Support Vector Machines^13^ and many more. These methods usually require efficient algorithms.

In addition, transcriptomics data frequently contain erroneous or noisy values. Independent of whether these values are caused by measurement errors or inherent outlying behavior, they can influence the classification process of all the remaining patients ^14^. Robustness to outliers can be achieved by robust methods which identify outliers (also denoted as influential samples) during the classification process. A novel approach for outlier detection by Lopes et al. ^15^, for example, applies a consensus approach that combines the inherent residual measures of several classification methods to obtain a consensus ranking of samples in terms of their outlierness. Since feature selection and also outlier detection methods are based on different assumptions, their performance also varies depending on the specific characteristics of the dataset which they are applied to. Likewise, a comparison of different methods in an in silico study also depends to a considerable extend on the model which has been used for data generation, since every method has its strengths and weaknesses and there is not a single best solution. The idea of Ensemble approaches is to combine several methods which return the same kind of output in order to increase accuracy and reduce the number of false positively selected features. It has already been shown that the sparse Ensemble approach of Lopes et al. ^15^ achieves high accuracy in feature selection compared to other sparse and robust classifiers in settings where the number of outliers is low ^16^. However, in datasets with a larger proportion of outliers, these might have an impact on the classification, and thus on the results of outlier detection and feature selection. Therefore, important features can be missed in the selection.

Combining the idea of an Ensemble approach with the need for robustness against outliers, we propose to use an Ensemble of robust sparse methods, which we name RObust Sparse ensemble for outlIEr detection and feature selection (ROSIE). The general workflow of ROSIE (i) combines sparse and robust classification methods for outlier detection and feature selection and (ii) performs a validity check in terms of altered data.

To build our Ensemble, we selected three sparse and robust methods with freely available implementations in R packages, to perform supervised (classification) and unsupervised (clustering) learning tasks: Sparse robust discriminant analysis with sparse partial robust M regression ^17,18^ (SPRM-DA or SPRM), Robust and sparse K-means clustering ^19,20^ (RSK-means), and Robust and sparse logistic regression with elastic net penalty ^21,22^ (enetLTS). For each method, a ranking of outlierness for all features is obtained and combined to a single consensus ranking by calculating the Rank Product (RP). Outlierness is subsequently assessed using the RP test. Bootstrap samples drawn from the original dataset are used to verify results.

This pipeline is evaluated on simulated data. Results show that the procedure identifies outliers reliably in different settings. Subsequently, ROSIE is applied to a transcriptomic breast cancer dataset to differentiate triple-negative breast cancer (TNBC) from other breast cancer types (non-TNBC). TNBC is an aggressive breast cancer subtype, with a marked heterogeneity and a poor survival, for which the selection of new biomarkers for the development of new targeted therapies is of clinical relevance ^23^. ROSIE is indeed able to select features in a robust way. Morover, several of the selected genes have been associated with TNBC in other experimental and machine learning contexts, which corroborates the biological significance of the genes selected by ROSIE.

## Methods

### Ensemble procedure

**Figure 1.**
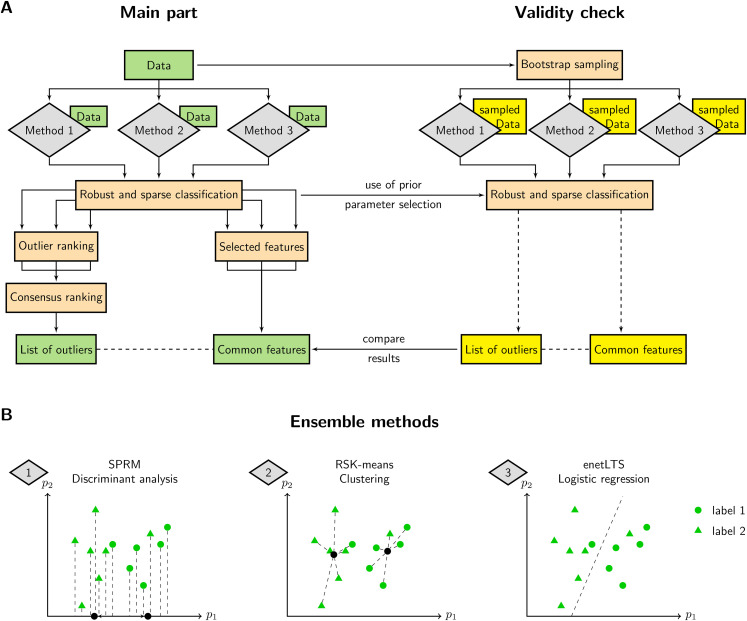
ROSIE workflow and robust and sparse classification methods. A) Three robust and sparse methods perform classification on the dataset. Each method provides an outlier ranking and selected features. Rankings are combined to acquire an outlier list. Important features are taken as the intersection of all three selected feature sets. Validity of the method is assessed by repeatedly classifying bootstrap sampled datasets and comparing the results with the main part. B) Simplified representation of the underlying classification methods, i.e., sparse robust discriminant analysis with sparse partial robust M regression (SPRM), robust and sparse K-means clustering (RSK-means) and robust and sparse logistic regression with elastic net penalty (enetLTS) for exemplary data comprising two classes and two features ( $p_{1},p_{2}$ ).

The Ensemble procedure is illustrated in Figure 1A. It can be divided into two parts. In the first and main part (Figure 1A (left)), three classification methods are applied independently from each other to the dataset. Hyperparameters for each method are optimized during this step. Since all methods are sparse and robust, each of them returns a list of selected features and a measure for the outlier ranking of the samples. Commonly selected features are marked as important. Moreover, using the RP test to achieve a consensus ranking, we finally obtain a list of outliers by evaluation of the corresponding q -values.

The second part (Figure 1A (right)) consists of a validity check which verifies the results of the main part with resampled data. For this purpose, several bootstrap sets are taken from the original dataset while preserving the dataset size and proportion of samples labeled with 0 and 1 , respectively. The classification methods are applied to these bootstrap samples using the optimal hyperparameters identified in the main part. The resulting lists of outliers and selected features are used to evaluate the results of the main part.

### Ensemble methods

We selected three inherently different methods for classification in order to obtain independent ranking results. A schematic depiction of the approaches with arbitrary data points of two classes is given in Figure 1B. A formal description of each method, the choice of hyperparameters, as well as the ranking of outliers and the selection of features are detailed in Supporting Information Section S1.

### Outlier identification

The identification of outliers by combining the results of different classifiers can in the simplest way be achieved by finding the intersection of samples tagged as outliers by each method. But not only do not all methods provide such tags, this procedure also does not have any statistical background. We therefore apply an Ensemble method based on the RP technique ^24^. This non-parametric statistical technique is based on the RP from different methods and permits the calculation of significance rankings for all samples. Therefore, as depicted in the Ensemble workflow (Figure 1A), we require the outlier rankings for each classification approach. As the classifiers differ in their procedure of classification and outlier detection, rankings are obtained in an individually adjusted fashion, as described for each of the methods. Independent of the ranking rule, an average approach (software settings ties.method = "average") is applied for tied values. Thus, for each sample i∈{1,…,n} we obtain three rank values $R_{l}(i),\;l\in \{1,\;2,\;3\}$ .

In order to combine these rankings to one consensus ranking, we calculate the RP for each individual as $RP(i)=\prod_{l=1}^{3}R_{l}(i)$ . Subsequently, samples are ranked according to their RP values. Corresponding p -values are then determined using the approach of Heskes et al. ^25^. Statistical testing of all p -values increases the risk of type I errors (false positives), since for each test a type I error can occur. In order to control the type I error in multiple testing, the expected proportion of type I errors among all significant test results, i.e., the False Discovery Rate (FDR) ^26,27^, can be considered. While a False Positive Rate of 5% implies that on average 5% of true null hypotheses are rejected, an FDR of 5% means that on average 5% of all rejected null hypotheses are actually true. As a measure of the FDR, so called q -values are calculated based on the p -values. q -values as measures of the FDR are the analogue of the p -values as measures of the False Positive Rate and provide a mechanism to control the rate of false discoveries in multiple tesing problems.

### Validity check

In order to assess the robustness of ROSIE towards variations in the data, we repeat the classification and evaluation steps for different alterations of the original data created by bootstrap sampling (see Figure 1A, right side). For m data variations, the samples are separated in m blocks of approximately equal size while keeping the proportion of the classes. Each block is subsequently filled to original size with data points that are sampled with replacement from the complete dataset. Again, we ensure preservation of the case proportion. This sampling strategy ensures that each sample is contained in at least one bootstrap block. In the next step, classification is performed for each block given the parameters that were selected in the main Ensemble run. Finally, the entirety of influential samples found in the bootstrap runs are compared with the influential samples of the main run. Likewise, we examine the match of selected features found in the main run and the bootstrap runs. In addition to validating our procedure, this approach can be used to reduce the number of features to be evaluated by considering only those that have been repeatedly selected also in the bootstrap runs.

### Simulation Study

In order to evaluate our ensemble compared to each individual method in a controlled setting in which the ground truth is known, we performed a simulation study on artificial data comprising 3200 features and 200 samples (as detailed in Supporting Information Section S3). Outliers were created in two different ways. First, a subgroup of samples was randomly selected and their labels switched. This reflects errors in the a priori classification. This was performed for 5% and 15% of the samples, respectively, leading to two datasets. Second, in order to mirror outliers in gene expression in the third dataset, 15% of the features were randomly selected and their standard deviation computed. Then, 5% of the samples were randomly selected and the values corresponding to the selected features increased by three times the respective standard deviation.

### Triple-negative breast cancer data/ Data preparation

We considered a dataset consisting of RNA sequencing (RNA-Seq) data of breast cancer patients from The Cancer Genome Atlas ^28^ Breast Invasive Carcinoma data collection. The Cancer Genome Atlas^29^ comprises one of the largest collections of omics datasets for more than 33 different cancer types and 20,000 individual tumour samples. The dataset used to evaluate ROSIE was the one used in Lopes et al. (2018) ^15^, corresponding to the Breast Invasive Carcinoma RNA-Seq Fragments Per Kilobase per Million (FPKM), excluding the clinical variables subset. The dataset was obtained using the brca.data R package ^30^, as described by the authors ^15^.

The dataset consists of 1,019 patients (samples) in total, of which 160 are TNBC (class membership $y_{j}=1$ ) and 859 non-TNBC ( $y_{j}=0$ ). The expression of three receptors was used to assign class labels to the samples. Patients are labeled as TNBC when the genes for the estrogen receptor and progesterone receptor are not expressed while the human epidermal growth factor receptor 2 (HER2) is not overexpressed.

HER2 measurements based on three different readouts were available for a classification of samples, HER2 (via immunohistochemical testing (IHC)) level, HER2 (via IHC) status and the HER2 level measured by fluorescence in-situ hybridization testing (FISH). Altogether, 28 patients showed non-concordance between two of the resulting HER2 labels, of which 4 were assigned to the TNBC and 24 to the non-TNBC group. We refer to these patients as *suspect* samples. For 8 out of these 28 suspect samples, HER2 decision also decides label. We note here that although the non-TNBC group consists of several subgroups, they are all assumed to be similar enough, such that binary classification is not hampered.

The huge amount of raw data was reduced by considering for the analysis only protein coding genes reported by the Ensembl genome browser ^31^ and the Consensus Coding Sequence project ^32^. By additionally removing genes whose expression level remained constant across all patients, a subset of 19,688 genes (features) was extracted. We further reduced the number of genes to 16,600 in a final step of data preparation, as SPRM is restricted in the data size it can process. Reduction was performed by employing the function filterVarImp from the R package caret ^33^, which performs class prediction for a series of feature subsets. For each subset sensitivity, specificity and subsequently the receiver operating characteristic (ROC) curve are computed. The area under the ROC curve is then used as the measure of variable importance. By sorting the features according to their variable importance, we discarded those with lowest variable importance, such that 16,600 features remained. Data was log transformed for further analysis.

## Results and discussion

### Simulation Study: ROSIE reliably detects outliers in different settings

**Figure 2.**
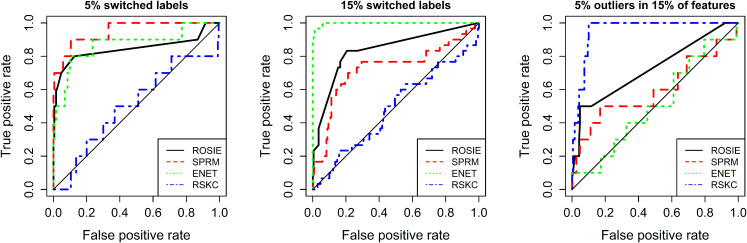
ROC curves for simulation study results. Results comparing ROSIE with single methods for three outlier settings. Average AUC values: ROSIE (0.81), ENET (0.79), SPRM (0.76), RSKC (0.65).

In order to investigate the performance of our procedure in detecting outliers, we applied ROSIE to the three simulated datasets. Details about the classification settings and the choice of the hyperparameters are given in Supporting Information S2 and S4. Results were compared with those of the individual approaches by ROC analysis (Figure 2). For the first dataset (Figure 2 left), SPRM performs best, tightly followed by enetLTS. Since RSKC is an unsupervised learning approach, which does not use the a priori labels, its performance is comparable to a random classification for the first and the second dataset, as expected. enetLTS outperforms the other approaches on the second dataset. Since ROSIE takes the outlier rankings of all three methods into account, it naturally cannot be the best method for a single dataset. However, its ROC curve is still acceptable even though RSKC completely fails in these particular scenarios. However, RSKC by far outperforms the other two methods on the third dataset, and enetLTS is not better than random. Also for this scenario, ROSIE still gives reasonable results. Moreover, ROSIE has the best overall AUC value when averaging over all three scenarios.

In summary, this analysis shows that the performance of the individual methods vary significantly and strongly depend on the particularly dataset at hand and the kind of outliers, while ROSIE is able to compensate for the failure of one of the methods. Moreover, on average, ROSIE detects outliers more reliably in terms of averaged AUC values. Since for real datasets the outlier percentage and noise levels are usually unknown a priori, ROSIE can indeed provide robust results in a situation of lack of detailed information.

### Breast cancer dataset: ROSIE selects features and outliers in a robust way

We examined the TNBC dataset with the three previously described methods SPRM, RSK-means and enetLTS. Details about the classification settings and parameter selections are given in the Supporting Information Section S2. Final parameter combinations for each method are listed in Table S3. Table 1 includes the number of selected genes and misclassifications for each of the methods. The methods result in similar numbers of misclassifications. A majority of 56 samples were commonly misclassified by all three methods (see Figure S1 for a Venn diagram of misclassified samples). The number of selected genes highly differs between the three methods, with differences up to two orders of magnitude. SPRM selects the largest number of genes, 2,982 , in the classification process. Interestingly, the 511 genes picked by RSK-means are a subset of this selection. Furthermore, only two of the 70 genes picked by enetLTS are not part of it. Taken together, a set of 54 genes was selected by all three methods (see Figure S2 for a Venn diagram of selected genes and Table S4 for a list of gene names). In summary, we have a remarkable agreement between the three methods regarding the set of misclassified samples as well as the set of selected genes.

**Table 1.** Summary of classification results. Number of selected features and number of misclassifications for SPRM, RSK-means and enetLTS.

	SPRM	RSK-means	enetLTS
# of selected genes	2,982	511	70
Misclassifications	68	63	63

After aggregating outlier rankings for all three methods and calculating the q -value for each sample, 11 samples with q<0.05 were identified as influential (Table 2). All influential samples are of type non-TNBC, while all but one of these samples are classified as TNBC by each of the three classification methods. Also, that one is still misclassified by two of the methods. This list shows that ROSIE has the potential to detect potential misclassifications also in cases where labels are initially missing. Furthermore, the list of misclassifications is enriched by suspect cases, which is further reassuring.

**Table 2.** Summary for influential samples found by Ensemble procedure. Shown are acquired ranks per method, Rank Product (RP), statistical p - and q -values, misclassification percentage and percentage of significant q -values in bootstrap runs. Suspect cases are marked with an asterisk (*). All influential samples were repeatedly selected as influential in all bootstrap runs they were included in.

	SPRM	RSK-m	enetLTS	RP	p -values	q -values	miscl. rate
TCGA-E9-A22G	1	94	1	94	0	0.0014	100
TCGA-A2-A0YJ	2	90	8	1440	0	0.0230	100
TCGA-A2-A4S1	61	1	43	2623	$1\cdot 10^{-4}$	0.0243	67
TCGA-A7-A13E	9	154	2	2772	$1\cdot 10^{-4}$	0.0243	100
TCGA-A2-A04U *	5	168	4	3360	$1\cdot 10^{-4}$	0.0243	100
TCGA-LL-A6FR	13	79	5	5135	$2\cdot 10^{-4}$	0.0296	100
TCGA-AR-A0TP	10	91	6	5460	$2\cdot 10^{-4}$	0.0296	100
TCGA-AR-A251	3	296	7	6216	$2\cdot 10^{-4}$	0.0299	100
TCGA-AN-A0FJ *	6	78	22	10296	$4\cdot 10^{-4}$	0.0410	100
TCGA-OL-A5S0	8	402	3	9648	$4\cdot 10^{-4}$	0.0410	100
TCGA-AN-A0FL *	39	13	24	12168	$5\cdot 10^{-4}$	0.0444	100

Five bootstrap samples were used to validate results. The three classification methods were applied to each of these samples, and commonly selected features and a list of influential samples were identified. A summary of the individual bootstrap optimization runs is given in Table S5 in the supplementary material. All influential samples were repeatedly selected as influential in all bootstrap runs they are part of. Influential samples appear in one up to all five of the bootstrap blocks with a mean appearance of 2.9 times. Moreover, 22 ( ≈41% ) of the commonly selected features of the main run are also commonly selected in all five bootstrap runs. Another 14 ( ≈26% ) are commonly selected in four bootstrap runs, while only three ( ≈6% ) are not commonly selected in any bootstrap run (see Table S4). Taken together, this analysis shows that ROSIE is able to select features and outliers in a robust way regarding variability in the data.

### Breast cancer dataset: Influential samples identified by ROSIE match well with the commonly selected genes

**Figure 3.**
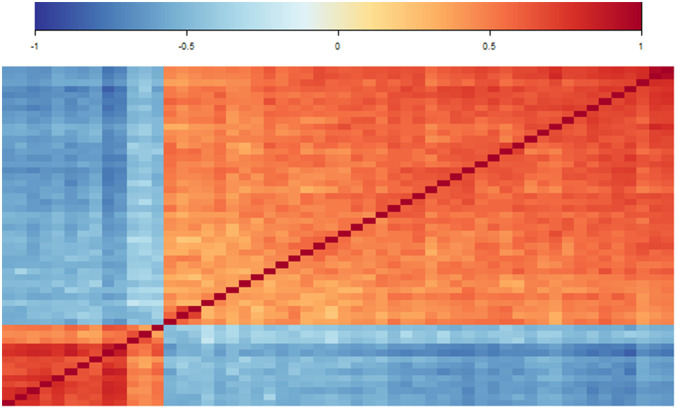
Correlation analysis of selected features. Heatmap of correlation values of the 54 commonly selected features.

In a first analysis step, we considered the correlation coefficients between the commonly selected genes. Figure 3 shows the corresponding heatmap of correlation coefficients. The genes show a clear separation into two blocks of predominantly moderate positive correlations while correlations between genes of different blocks are predominately moderate negative. The smaller block consists of 13 genes that are known to be downregulated in TNBC, for example *AGR2*, *TBC1D9* and *TGFB3*. The larger block comprises 41 genes that show upregulated behavior in TNBC samples, for example *FOXC1*, *UGT8* and *HORMAD1* (block membership of all 54 genes is noted in Table S4). Among commonly selected genes, absolute values of correlation coefficients range from 0.22 to 0.95 . In comparison, Figure S3 presents a corresponding heatmap of 54 randomly selected genes from the full dataset. Here, no such interrelated groups can be identified, and weak correlations dominate.

As the correlation values hint to a strong connection among the selected genes, we examined possible distinctive behavior of TNBC, non-TNBC and influential samples via density estimates.

**Figure 4.**
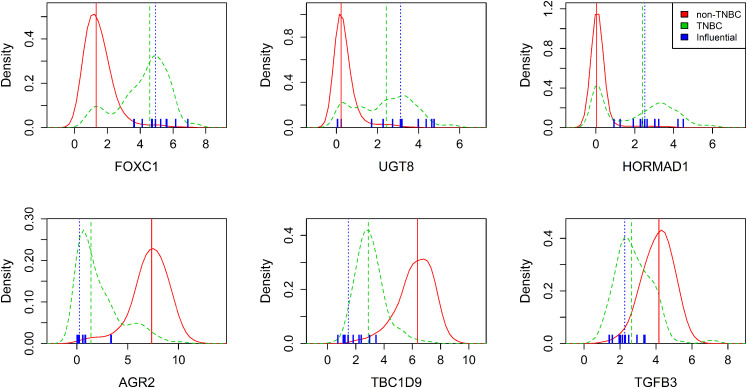
Relation between influential samples and commonly selected genes. Estimated densities of gene expression of selected features grouped by TNBC (green dashed line) and non-TNBC (red line). Vertical lines represent respective group medians. Blue markers depict influential samples.

For this purpose, we estimated two 1D marginal densities of commonly selected genes using all but the influential samples that were labeled as TNBC and non-TNBC, respectively, according to the TNBC markers, as described before. Figure 4A shows such density estimates exemplarily for six commonly selected genes. Density estimates of the non-TNBC group are represented by the solid red lines, respective estimates of the TNBC group are represented by the green dashed lines. Vertical lines illustrate the medians of both groups. In general, the densities of the non-TNBC group, which comprises around 84% of all samples, are for many genes close to normal distributions, while shapes of densities of the TNBC group vary substantially.

Densities partially show good separation between TNBC and non-TNBC groups, such as *FOXC1*, *AGR2* and *TBC1D9*. Here, the TNBC groups can roughly be summarized as right skewed or left skewed curves, respectively, with a median far from the non-TNBC median. In contrast, *HORMAD1* shows two distinct peaks for the TNBC group, one of which is in good agreement with the non-TNBC peak.

Along with this, values of markers for samples returned as influential by ROSIE are depicted in blue. They all were assigned to the non-TNBC group according to their markers prior to the classification approach. It can be seen that they strongly match the density curves of the TNBC group in all six plots and, related to that, they are distributed closely around the TNBC median. Particularly for *HORMAD1*, the influential sample values tend to have larger gene expression values, and thus fit particularly to the higher TNBC mode. Also, the median for influential and TNBC samples is very similar regarding *HORMAD1*.

The density plots for *UGT8* show another possible behavior of TNBC samples. Here, TNBC samples are rather uniformly distributed over a wide range of values that overlaps with the non-TNBC curve. Influential individuals are also widely spread, but the median still aligns with the TNBC samples.

Finally, *TGFB3* shows two overlapping curves with a seemingly bad separation of TNBC and non-TNBC. Still, the influential samples tend away from the non-TNBC peak and spread around the TNBC median instead.

Since density curves may not properly reflect the fact that around 84% of the samples are non-TNBC and the sample size for TNBC is comparably small, we present histograms of the TNBC and non-TNBC groups in Figure S4. Overall, Figure 4A shows that the influential samples selected via the RP test match well with the commonly selected genes, which in turn supports the potential of our ROSIE approach to identify important features and influential samples.

Based on these results, we asked the question whether the selected genes are primarily those which are differentially expressed between the two groups. Therefore, we applied edgeR (^37^, version 3.26.8) to the dataset in order to identify differentially expressed genes. In total, 7529 genes were found to be differentially expressed by this analysis. There is a very good agreement between the two methods. In particular, all genes found by ROSIE are among the differentially expressed genes identified by edgeR, thus reassuring that these are indeed correlated with the classification. Moreover, all those genes have a quite low false discovery rate, as can be seen by a ROC analysis with the genes found by ROSIE as ground truth (Figure S5). This analysis shows that ROSIE is able to identify DEGs as important features.

**Figure 5.**
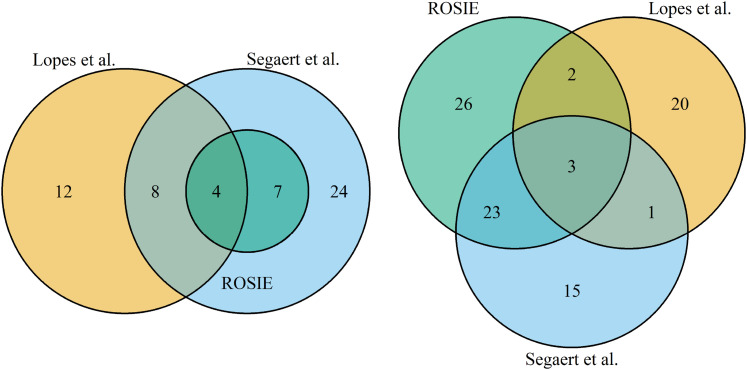
Venn diagrams comparing different classification approaches. Comparison of identified outliers (left) and selected genes (right) from ROSIE, the sparse Ensemble approach by Lopes et al. ^15^ and the robust approach enetLTS by Segaert et al. ^34^.

Analysis on influential samples and potential biomarkers on the TCGA dataset was also conducted by Lopes et al. ^15^ and Segaert et al. ^34^. The concordance of the three approaches are illustrated in the Venn diagrams in Figure 5. Lopes et al. used an Ensemble approach of sparse classification methods to identify outliers in the TCGA dataset using the RP statistics. 24 influential samples were identified, four of which coincide with our findings. The large difference in the number of outliers found by Lopes et al. and ROSIE is probably due to the fact that the three methods that were used in their ensemble approach are much more similar than in our approach.

Conversely, Segaert et al. used a single robust and sparse method, enetLTS, for outlier detection. Their results comprise 43 influential samples which include all of our findings. Both publications also present a set of genes as potential biomarkers for TNBC. Five genes which we identified as potential biomarkers were also found by the sparse Ensemble ^15^, while 26 are in common with Segaert et al. ^34^. Overall, this shows that our results are in line with other independent studies on the same dataset and additionally provide novel genes as potential putative biomarkers.

### Breast cancer dataset: Genes selected by ROSIE are associated with TNBC types in other studies

As the goal of this study is to show the capability of ROSIE for identifying biomarkers and influential samples in oncology data, we exemplarily investigate the biological background of three of the 54 selected genes. In the following, we will thus illustrate the significance of our findings by discussing the biological importance of the genes *HORMAD1*, *AGR2* and *TBC1D9* for TNBC, which presented especially strong indications of importance in literature.

HORMA domain containing 1 (*HORMAD1*) is one of the genes repeatedly selected also in the bootstrap runs of the Ensemble procedure. As HORMA domains play a role in chromatin binding, the protein encoded by *HORMAD1* has been suggested to be involved in meiosis and its expression as a potential marker for cancer ^35^. In previous studies analyzing differentially expressed genes between TNBC and non-TNBC, *HORMAD1* has already been highlighted as one of the key upregulated genes differentiating TNBC and non-TNBC ^36,38^. Additionally, *HORMAD1* overexpression, referring to the higher *HORMAD1* levels of the second mode of TNBC samples, has also been reported to contribute to Homologous Recombination Deficiency and to be a potential composite predictive biomarker for sensitivity to platinum-based chemotherapy in TNBC patients ^39^.

Similarly, *AGR2*, which has also been repeatedly selected in the Ensemble procedure, was listed among the top downregulated genes differentially expressed between TNBC and non-TNBC ^36,38^. It has been shown that *AGR2* is coexpressed with the estrogen receptor in breast cancer cell lines ^40^. In addition, it has been associated with cell migration and metastasis ^41^.

Finally, *TBC1D9* is a gene whose function has only recently been revealed to be involved in the regulation of selective autophagy via regulating *TBK1* activation, which in turn is often associated with cancer ^42^. Another recent study employed machine learning algorithms and survival outcome of breast cancer patients to identify three potential genes for the discrimination between TNBC and non-TNBC ^43^. Thereby, *TBC1D9* was selected, and overexpression of *TBC1D9* was furthermore shown to be connected to a better prognosis ^43^.

These aspects reinforce our findings of genes important for TNBC classification and the importance of identifying outlying individuals whose unique gene markup might influence their prognosis and drug sensitivity.

## Conclusions

In this study, we have presented ROSIE, a robust and sparse Ensemble approach for outlier detection and feature selection from high dimensional datasets. ROSIE combines different robust and sparse methods which are individually applied to the dataset. Thereby, hyperparameters are adjusted individually for each method, and a ranking of outliers as well as a set of selected features are defined. ROSIE combines these results into a consensus ranking by evaluating the q -values via the RP test, and by defining the set of selected features as features commonly selected by all methods. A validity check is done via a bootstrap approach. ROSIE was validated on simulated datasets and subsequently applied to RNA-Seq data from the TCGA for classification into TNBC and non-TNBC tissue samples.

Applying our Ensemble approach, we managed to reduce a set of 16,600 genes to 54 possible biomarkers for TNBC. ROSIE was able to identify features and outliers in a robust way. Furthermore, the identified set of potential biomarkers seems promising, since several of those genes also appear in other studies on differently expressed genes between TNBC and non-TNBC.

A survival analysis that compared TNBC cases, non-TNBC cases and outliers shows that outliers are all censored at early time points (Figure S6). In our opinion, this does not allow for any conclusions regarding similarity or dissimilarity between non-TNBC and outliers. If outliers were similar to the other class (here TNBC) in this analysis, one could argue that this probably hints to just a wrong labeling of those samples. However, this is not the case and needs further investigation in the future.

The workflow which we have presented can also be applied to other datasets with a large feature space and a low number of samples. In particular, it can handle outliers in the dataset. Overall, compared to the application of a single robust and sparse method, Ensemble approaches that combine inherently different methods might be superior in distinguishing spurious from true findings.

In future work, it remains to be seen how much the results of our Ensemble approach depend on the individual methods which are combined. In our application study, for example, we have observed a large similarity between SPRM and enetLTS, which overshadows the ranking results of RSK-means. As K-means can be seen as a special case of tclust ^14^, a more flexible robust clustering approach which is particularly designed to fit clusters with different scatters and weights, it would for instance be interesting to replace trimmed k-means by this more general approach blueor even more advanced versions ^44,45^ in future applications.

Furthermore, ROSIE suffers from long run times, especially for RSK-means, which has the longest run time despite the smallest number of parameter combinations in the parameter selection step. This needs to be addressed to make ROSIE applicable to larger datasets in future work.

## Supplemental Material

sj-pdf-1-smm-10.1177_09622802211072456 - Supplemental material for ROSIE: RObust Sparse ensemble for outlIEr detection and gene selection in cancer omics data Click here for additional data file. Supplemental material, sj-pdf-1-smm-10.1177_09622802211072456 for ROSIE: RObust Sparse ensemble for outlIEr detection and gene selection in cancer omics data by Antje Jensch, Marta B. Lopes, Susana Vinga and Nicole Radde in Statistical Methods in Medical Research

## Abbreviations

enetLTS: Robust and sparse logistic regression with elastic net penalty
FDR: False Discovery Rate
HER2: human epidermal growth factor receptor 2
IHC: immunohistochemical testing
LDA: linear discriminant analysis
RNA-Seq: RNA sequencing
ROSIE: RObust Sparse ensemble for outlIEr detection and feature selection
RP: Rank Product
RSK-means: parse robust discriminant analysis with sparse partial robust M regression
SPRM/SPRM-DA: Sparse robust discriminant analysis with sparse partial robust M regression
TCGA: The Cancer Genome Atlas
TNBC: Triple-Negative Breast Cancer
