# Supplementary material for: ROSIE: RObust Sparse ensemble for outlIEr detection and gene selection in cancer omics data
Source: Stat Methods Med Res. 2022 Jan 24;31(5):947–58. doi: 10.1177/09622802211072456 (PMC9014683; doi:10.1177/09622802211072456)
Supplement: sj-pdf-1-smm-10.1177_09622802211072456 - Supplemental material for ROSIE: RObust Sparse ensemble for outlIEr detection and gene selection in cancer omics data [file sj-pdf-1-smm-10.1177_09622802211072456.pdf]

ROSIE: RObust Sparse Ensemble for outlIEr detection and gene selection in cancer omics data

Antje Jensch, Marta Lopes, Susana Vinga and Nicole Radde

Supporting Information

1 Classification methods

For a short formal description of each method, let the data consist of a sample set $\boldsymbol{X} \in \mathbb{R}^{n\times p}$, the predictor, with n samples $\boldsymbol{x}_i = (x_{i,1}, \dots, x_{i,p})$, $i = 1, \dots, n$ and p features as well as the response vector $\boldsymbol{y}$, a binary vector of length n that encodes the class membership.

1.1 Sparse robust discriminant analysis with sparse partial robust M regression (SPRM-DA)

Sparse robust discriminant analysis with sparse partial robust M regression [1] (SPRM-DA, in the following SPRM) classifies samples $\boldsymbol{x}_i$ by maximizing the distance between group means and minimizing the variance within groups on a projected hyperplane. Therefore, the response vector $\boldsymbol{y}$ is given in centered and scaled form and further treated as continuous variable. Likewise, the matrix $\boldsymbol{X}$ is column-wise centered. SPRM consists of two parts. In the first part, based on partial least squares [2], the data $\boldsymbol{X}$ is reduced by projecting it to a lower dimensional subspace $\mathbf{XW} \in \mathbb{R}^{n\times H}$, $H < p$, where $\mathbf{W} \in \mathbb{R}^{p\times H}$ is characterized by direction vectors $\boldsymbol{w}_h \in \mathbb{R}^p$, $h = 1, \dots, H$ and $H < p$ defines the reduced dimensionality of the subspace. This is achieved by maximizing the squared covariance between the predictor projection $\boldsymbol{Xw}$ and the response vector $\boldsymbol{y}$,

$$\boldsymbol{w}_h = \arg\max_{\boldsymbol{w}} \operatorname{cov}^2(\boldsymbol{Xw}, \boldsymbol{y}) \tag{1}$$

for $h \in \{1, \dots, H\}$ subject to $\|\boldsymbol{w}_h\| = 1$ and $\boldsymbol{w}_h^T \boldsymbol{X}^T \boldsymbol{X} \boldsymbol{w}_i = 0$ for $1 \leq i < h$. Many algorithms have been suggested to solve problem (1). The one that is used in [1] makes use of the standard covariance estimator

$$\widehat{\operatorname{cov}}^2 = \left(\frac{1}{n-1} \boldsymbol{y}^T \boldsymbol{Xw} \right)^2 . \tag{2}$$

In a second step, the transformed data is classified using Fisher's linear discriminant analysis (LDA).

Robust DA with partial robust M regression uses the concept of M estimation as a powerful tool in robust statistics to identify outliers. Therefore, weights between 0 and 1 are assigned to each sample to regulate its influence on model estimation. Weights are chosen such that samples $\boldsymbol{x}_i$ with large distances with respect to the center and covariance of its assigned class, quantified by a weighting function on the robust squared Mahalanobis distance, are downweighted. These weights enter both steps of the procedure, i.e., covariance maximization, where the weights are determined iteratively, and LDA, where the optimized weights are used to perform a weighted, robust LDA. The procedure how optimal weights are achieved is explained in [1]. Integration of these weights modifies the optimization problem (1) to

$$\hat{\boldsymbol{w}}_h = \arg\max_{\boldsymbol{w}} \operatorname{cov}^2(\boldsymbol{X}_{\boldsymbol{\Omega}} \boldsymbol{w}, \boldsymbol{y}_{\boldsymbol{\Omega}}), \tag{3}$$

where $\boldsymbol{\Omega} = \operatorname{diag}(\omega_1, \dots, \omega_n)$ downweights samples, leading to weighted data matrix and response vector $\boldsymbol{X}_{\boldsymbol{\Omega}} = \boldsymbol{\Omega X}$ and $\boldsymbol{y}_{\boldsymbol{\Omega}} = \boldsymbol{\Omega y}$, respectively. Constraints for $\boldsymbol{w}_h$ apply accordingly.

In addition, sparsity is ensured by penalizing the estimation of the direction vectors $\boldsymbol{w_h}$ with an ℓ_1 norm penalty η. This regularization forces complete rows of the weight matrix $\boldsymbol{W}$ to become zero, and the respective features have no influence. Thus, only features with nonzero weights are selected. The resulting optimization problem is described in [1] (equations (18)).

Hyperparameters of SPRM include the number H of latent components and the sparsity parameter η, which are determined by a cross-validation procedure as described in [1].

We employ the sample weights, as provided by the classification output, as a measure for outlier ranking in the outlier ranking step in Figure 1A in the main manuscript. Samples are thus ranked in ascending order of their weights. The selected features are extracted as the set of features that have at least one non-zero entry in the corresponding row of the weight matrix $\boldsymbol{W}$.

1.2 Robust and sparse K-means clustering (RSK-means)

The second employed classifier is Robust and sparse K-means clustering (RSK-means) [3]. RSK-means is based on the standard K-means clustering [4], which searches for a partition of the dataset into K clusters by minimizing the within-cluster sum of squares or, equivalently, maximizing the between-cluster sum of squares. If a large fraction of features are not related to the response variables and only few features contribute to the differences between samples in different clusters, K-means often fails. This problem was first addressed by Witten and Tibshirani [5], who proposed sparse K-means to simultaneously find clusters and a small number of features which are sufficient to unravel the cluster structure. This is achieved by assigning weights $\boldsymbol{w} = (w_1, \ldots, w_p), w_j \geq 0,\ j = 1, \ldots, p$ to each feature that are constraint in their norms to enforce sparsity, leading to the optimization problem

$$\max_{C_1,\ldots,C_k,\boldsymbol{w}} \sum_{j=1}^{p} w_j \left\{ \frac{1}{n} \sum_{i=1}^{n} \sum_{i'=1}^{n} d_{i,i',j} - \sum_{k=1}^{K} \frac{1}{n_k} \sum_{i,i' \in C_k} d_{i,i',j} \right\} \tag{4}$$

subject to $\|\boldsymbol{w}\|_2 \leq 1$ and $\|\boldsymbol{w}\|_1 \leq l$. Here, $l > 1$ determines the degree of sparsity in terms of non-zero weights of the solution, and $C_1, \ldots, C_K$ denotes the partition into K different clusters. In equation (4), the expression in the brackets describes the between-cluster sum of squares, with additive dissimilarity measure $d_{i,i'} = \sum_{j=1}^{p} d_{i,i',j}$ between samples i and i', which can, e.g., be chosen as squared Euclidean distance between $\boldsymbol{x}_i$ and $\boldsymbol{x}_{i'}$, i.e., $d_{i,i',j} = (x_{i,j} - x_{i',j})^2$. The variable n_k denotes the number of individuals in cluster k.

Like K-means clustering, problem (4) is in practice solved by iterating the following steps:

1. Given weights $\boldsymbol{w}$ and cluster centers $\boldsymbol{\mu}_1, \ldots, \boldsymbol{\mu}_K$, assign samples to the cluster with the closest center in terms of weighted Euclidean squared distances.

2. Based on this partitioning, update cluster centers to the weighted sample means of the samples in the respective clusters.

3. Choose weights subject to constraints such that the weighted between cluster sum of squares is maximized.

Following the idea of Cuesta-Albertos and Gordaliza [6] to achieve a clustering that is robust to outliers by trimming α 100% of the samples with largest distances to their cluster centers in step 2, Kondo et al. [3] introduced a modified algorithm, RSK-means, which combines SK-means with a trimming procedure which finally returns a set of selected features as well as a set of outliers. Thereby, the final set of outliers O is obtained as the union of the sets O_W and O_E, which are calculated with and without weights, respectively. Optimal weights $\boldsymbol{w}$ are determined by maximizing the between-cluster sum of squares under exclusion of observations flagged as outliers in the set O,

$$\max_{\|\boldsymbol{w}\|_2 \leq 1, \|\boldsymbol{w}\|_1 \leq l} \sum_{j=1}^{p} w_j \left[\frac{1}{n - |O|} \sum_{i=1}^{n-|O|} \sum_{i'=1}^{n-|O|} d_{i,i',j} - \sum_{k=1}^{K} \frac{1}{n_{k,O}} \sum_{i,i' \in C_{k,O}} d_{i,i',j} \right]. \tag{5}$$

Here, $C_{k,O}$ represents the truncated k-th cluster and $n_{k,O}$ the corresponding number of samples.

RSK-means requires the selection of three hyperparameters, the L_1 bound l, which determines the degree of sparsity and can be chosen to achieve a desired number of selected features, the trimming proportion α, and the number of clusters K. In order to select l and α, classification runs for different combinations of parameter values were performed. Final parameters were selected as best combination with respect to the classification error rate as provided by the CER function from the RSKC[7] package. Ranges for each parameter are given in Table S1. We defined $K = 2$ in accordance to the binary response vector $\mathbf{y}$ supplied in the classification process.

For the outlier ranking step in Figure 1A in the main manuscript, we calculate the Euclidean distance of cases from the cluster center using the cluster partition obtained by the classifier. Cluster centers are determined without identified outliers O and including feature weights

$$D_i(\boldsymbol{x}_i) = \sum_{j=1}^{p} w_j (x_{i,j} - \mu_{k,j})^2, \tag{6}$$

for $i \in C_k$ and $\mu_{k,j} = \frac{1}{n_{k,O}} \sum_{i \in C_{k,O}} x_{i,j}$, $k \in \{1, 2\}$. Since larger distances from the cluster center correspond to a higher chance of being an outlier, ranking is assigned in descending order of distance D_i. Furthermore, the features weights w_j, $j = 1, \ldots, p$, are evaluated. Features with corresponding non-zero weights constitute the set of selected features.

1.3 Robust and sparse logistic regression with elastic net penalty (enetLTS)

Robust and sparse logistic regression with elastic net penalty (enetLTS)[8] uses a logistic regression model to determine a regression hyperplane between the groups. Therefore, the logistic regression model $y_i = \pi_i + \varepsilon_i$, for $i = 1, \ldots, n$, is used to describe the relation between the predictor $\boldsymbol{X}$ and the response $\boldsymbol{y}$. The term ε_i describes a binomially distributed error, and π_i denotes the conditional probability for the i-th individual to belong to class one,

$$\pi_i = P(y_i = 1 | \boldsymbol{x}_i) = \frac{\exp(\boldsymbol{x}_i^T \boldsymbol{\beta})}{1 + \exp(\boldsymbol{x}_i^T \boldsymbol{\beta})}, \tag{7}$$

with regression coefficients $\boldsymbol{\beta} \in \mathbb{R}^p$. In case $n > p$ optimal regression coefficients $\hat{\boldsymbol{\beta}}$ are identified by minimizing a deviance function $d(\boldsymbol{x}_i^T \boldsymbol{\beta}, y_i)$,

$$\hat{\boldsymbol{\beta}} = \arg\min_{\boldsymbol{\beta}} \sum_{i=1}^{n} d(\boldsymbol{x}_i^T \boldsymbol{\beta}, y_i) = \arg\min_{\boldsymbol{\beta}} \sum_{i=1}^{n} -y_i \boldsymbol{x}_i^T \beta + \log\left(1 + e^{\boldsymbol{x}_i^T \boldsymbol{\beta}}\right). \tag{8}$$

The method is adjusted for multicollinearity among the predictors and cases of $n < p$ by adding an elastic net penalty term

$$P_\alpha(\boldsymbol{\beta}) = (1-\alpha)\frac{1}{2}\|\boldsymbol{\beta}\|_2^2 + \alpha\|\boldsymbol{\beta}\|_1 = \sum_{j=1}^{p}\left[(1-\alpha)\frac{1}{2}\beta_j^2 + \alpha|\beta_j|\right] \tag{9}$$

to equation (8),

$$\hat{\boldsymbol{\beta}}_{\text{enet}} = \arg\min_{\boldsymbol{\beta}} \left\{ \sum_{i=1}^{n} d(\boldsymbol{x}_i^T \boldsymbol{\beta}, y_i) + \lambda P_\alpha(\boldsymbol{\beta}) \right\}. \tag{10}$$

The tuning parameter $\lambda \geq 0$ determines the strength of the penalty and thus sparsity, and $\alpha \in [0, 1]$ defines the mixing proportion of the ℓ_1 and ℓ_2 norm. In addition, the method becomes robust against outliers by iteratively trimming the sample set to an optimal subset

$$\hat{\boldsymbol{\beta}}_{\text{enetLTS}} = \arg\min_{\boldsymbol{\beta}, H} Q(H, \boldsymbol{\beta}) = \arg\min_{\boldsymbol{\beta}, H} \left\{ \sum_{i \in H} d(\boldsymbol{x}_i^T \boldsymbol{\beta}, y_i) + h\lambda P_\alpha(\boldsymbol{\beta}) \right\}, \tag{11}$$

where $H \subseteq \{1, 2, \ldots, n\}$ with $|H| = h$. This subset is supposed to be outlier free, and hence all individuals that are not contained in the subset are defined as outliers. Solving problem (11) is in general a difficult

problem, which is solved in an iterative way. First, an optimal set $H_{opt} = \arg\min_{H \subseteq \{1,\ldots,n\}, |H|=h} Q(H, \hat{\boldsymbol{\beta}}_H)$ is found as explained in[8]. Then, optimal regression parameters $\hat{\boldsymbol{\beta}}_{\text{enetLTS}}$ are found via optimizing the objective function $Q(H_{opt}, \boldsymbol{\beta})$ with respect to $\boldsymbol{\beta}$.

Altogether, enetLTS requires the selection of three hyperparameters. These consist of α, which describes the mixing proportion of the two penalty terms in equation (9), λ defining the strength of the penalty and thus the degree of sparsity, as well as the subset proportion h_p. For the optimization, the original set of n samples is reduced by the proportion h_p, resulting in the trimmed set of size $h = h_p \cdot n$. Cross validation for different combinations of values of α, λ and h_p is performed. The range of values is presented in Table S1.

In order to obtain an outlier ranking, we first calculate the absolute value of the Pearson residual r_i, $i \in \{1, \ldots, n\}$,

$$r_i = \left| \frac{y_i - \pi_i}{\sqrt{\pi_i(1 - \pi_i)}} \right| \tag{12}$$

with π_i from Equation (7). Following the same reasoning as for RSK-means, we then rank from largest to smallest residual. Finally, the set of selected features is formed by all features with non-zero coefficient $\hat{\beta}_{\text{enetLTS},j}$, $j = 1, \ldots, p$.

2 Classification setup

All computations were performed on R version 3.3.3[9].

We applied the three classification methods on the reduced dataset using default settings for enetLTS and RSK-means. However, for SPRM we specified scale = standard deviation, center = mean and fun = Fair as weighting function for the case weights.

Optimal parameters were chosen according to cross validation of a range of values for all parameters for enetLTS and SPRM, as implemented in the corresponding packages.

Table S1: Parameter ranges for parameter selection

Method	Parameter	Range for parameter selection
SPRM	α	{1, 2, ..., 5}
	η	{0.2, 0.3, ..., 0.9}
RSK-means	α	{0.05, 0.1, 0.15, 0.2}
	l	{15, 16, ..., 20}
enetLTS	α	{0.1, 0.2, ..., 0.8}
	λ	{0, 0.05, ..., 0.2}
	h_p	{0.7, 0.75, ..., 0.9}

3 Simulation study

From the entire set of genes of the breast cancer dataset, a subset consisting of 3200 genes was selected as features. Since the simulation study was conducted after applying ROSIE to the breast cancer dataset, we decided to include the 54 commonly selected genes. Thus, the 54 commonly selected genes were chosen a priori and the remaining number was filled up randomly. Means and covariances were then computed for the resulting dataset for each the TNBC and non-TNBC groups. In order to achieve a similar class ratio as in the original dataset (160 TNBC vs. 859 non-TNBC, which corresponds to about 16% TNBC), we drew about 16% (31) samples from a multivariate normal distribution with mean and covariance matrix calculated for the TNBC group and about 84% (169) samples from a multivariate normal distribution with mean and covariance matrix calculated for the non-TNBC group. These datasets were corrupted by outliers as description in the main manuscript.

4 Additional tables and figures

Table S2: **Hyperparameters.** Optimal parameters found by cross-validation for the simulation study.

	SPRM	RSK-means	enetLTS
5% switched labels	$\alpha = 2$<br>$\eta = 0.5$	$\alpha = 0.05$<br>$l = 14$	$\alpha = 0.2$<br>$\lambda = 0.05$<br>$h_p = 0.85$
15% switched labels	$\alpha = 1$<br>$\eta = 0.7$	$\alpha = 0.05$<br>$l = 18$	$\alpha = 0.2$<br>$\lambda = 0.05$<br>$h_p = 0.7$
5% outliers in 15% of features	$\alpha = 1$<br>$\eta = 0.5$	$\alpha = 0.05$<br>$l = 14$	$\alpha = 0.1$<br>$\lambda = 0.05$<br>$h_p = 0.7$

Table S3: **Hyperparameters.** Optimal parameters found by cross-validation for the breast cancer dataset.

	SPRM	RSK-means	enetLTS
Parameters	$\alpha = 1$<br>$\eta = 0.4$	$\alpha = 0.05$<br>$l = 16$	$\alpha = 0.6$<br>$\lambda = 0.05$<br>$h_p = 0.75$

Table S4: List of commonly selected genes sorted according to the number of common selections in bootstrap runs. Bold names represent genes at least partially downregulated in TNBC samples (smaller block of positively correlated genes in Figure 3 in the main manuscript), while remaining genes are at least partially upregulated (larger block of positively correlated genes in Figure 3 in the main manuscript).

5×	4×	3×	2×	1×	0×
FOXA1	***GATA3***	***CA12***	***TBC1D9***	*RPIA*	***TGFB3***
SPDEF	*DLX6*	***CAPN13***	***GALNT10***	*FABP7*	***AGR3***
MLPH	*SOX8*	*OTX1*	***CMBL***	*C16orf95*	*FAM136A*
CXXC5	*SOX6*	*GCNT2*	*STAC*		
AGR2	*CHRM3*	*PPP1R14C*	*MELTF*		
OCA2	*TMCC2*		*MICALL1*		
VGLL1	*A2ML1*		*TTLL4*		
ROPN1B	*UGT8*				
ROPN1	*CDCA2*				
FOXC1	*LEMD1*				
PAPSS1	*SMOC1*				
HORMAD1	*POU5F1*				
ZIC1	*SFT2D2*				
SRSF12	*NKX1-2*				
CHODL					
ART3					
EN1					
TTYH1					
COL9A3					
FAM19A3					
FZD9					
CT83					

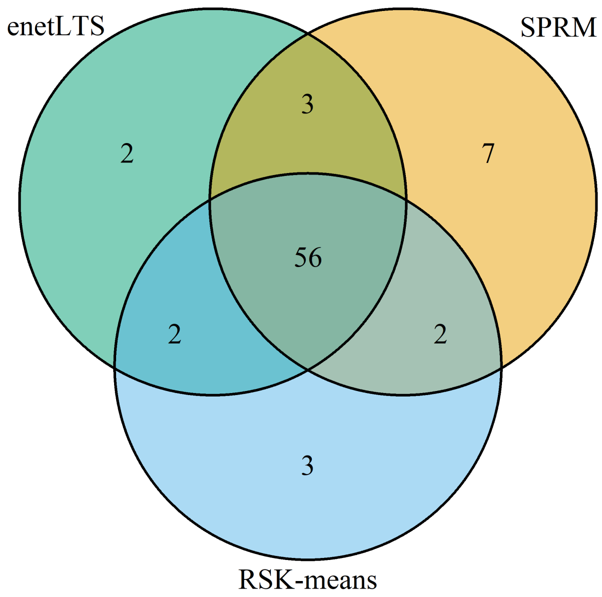

Fig S1: Venn diagram of samples misclassified by SPRM, RSK-means and enetLTS.

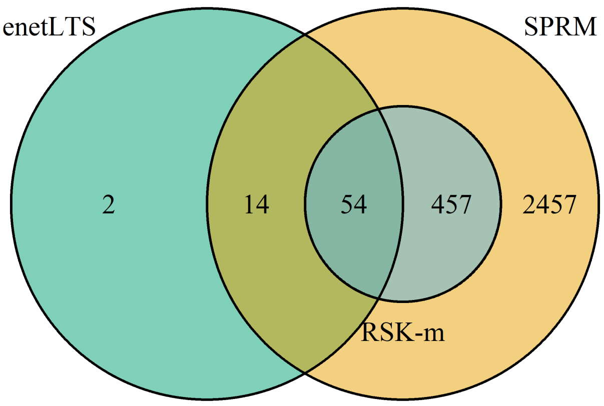

Fig S2: Venn diagram of the genes selected by SPRM, RSK-means and enetLTS.

Table S5: Summary of classification results for bootstrapped data including number of selected features and number of misclassifications for SPRM, RSK-means and enetLTS.

	SPRM	RSK-means	enetLTS	# of commonly selected features	# of influential samples
**Block 1**				71	13
# of selected genes	3206	505	210		
Misclassifications	67	65	50		
**Block 2**				83	11
# of selected genes	2902	519	164		
Misclassifications	50	57	41		
**Block 3**				81	13
# of selected genes	3193	511	187		
Misclassifications	62	61	169		
**Block 4**				82	13
# of selected genes	2857	522	222		
Misclassifications	68	67	160		
**Block 5**				82	11
# of selected genes	3095	517	211		
Misclassifications	60	61	44		

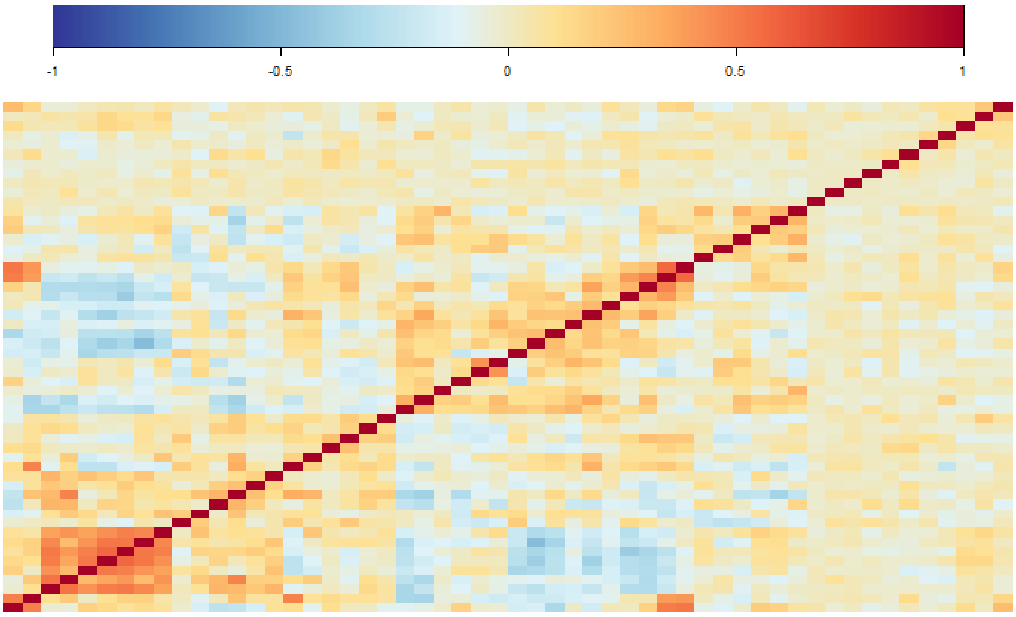

Fig S3: Heatmap of correlation values of 54 randomly selected features.

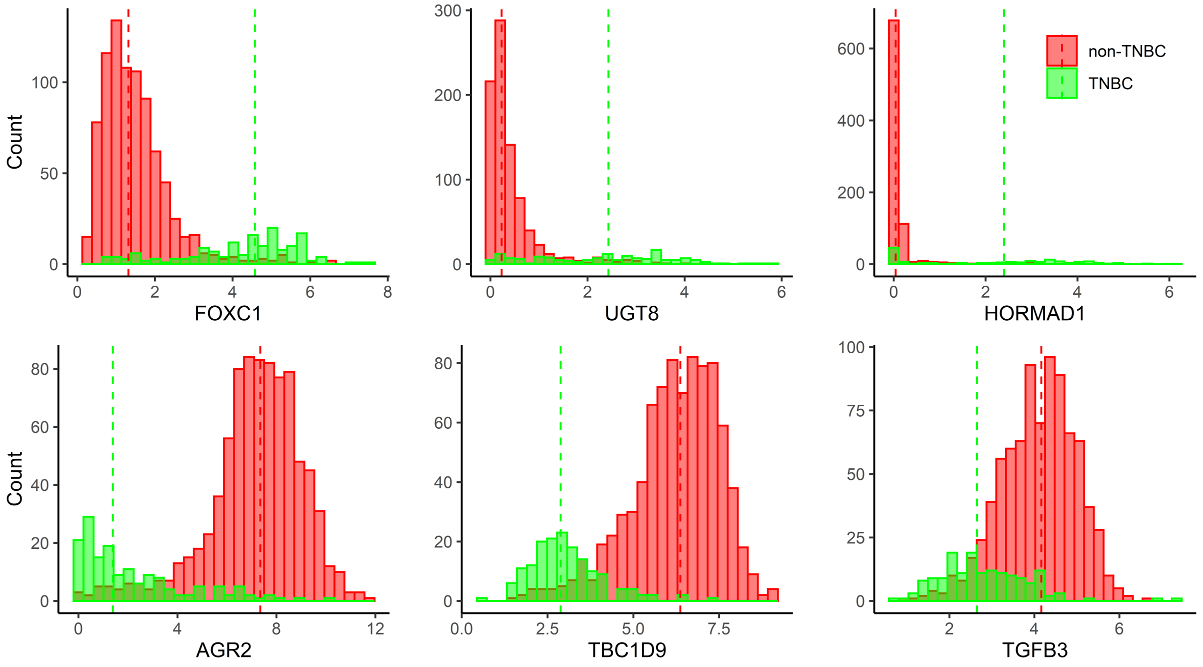

Fig S4: Groupwise histograms of TNBC (green), and non-TNBC (red) samples. Vertical lines represent respective group median.

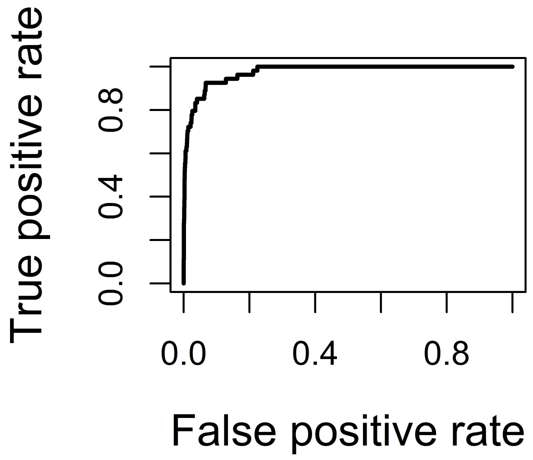

Fig S5: ROC curve analysis comparing differentially expressed genes found by edgeR with commonly selected genes from ROSIE. Cutoff values for ROC analysis were taken from the false discovery rate.

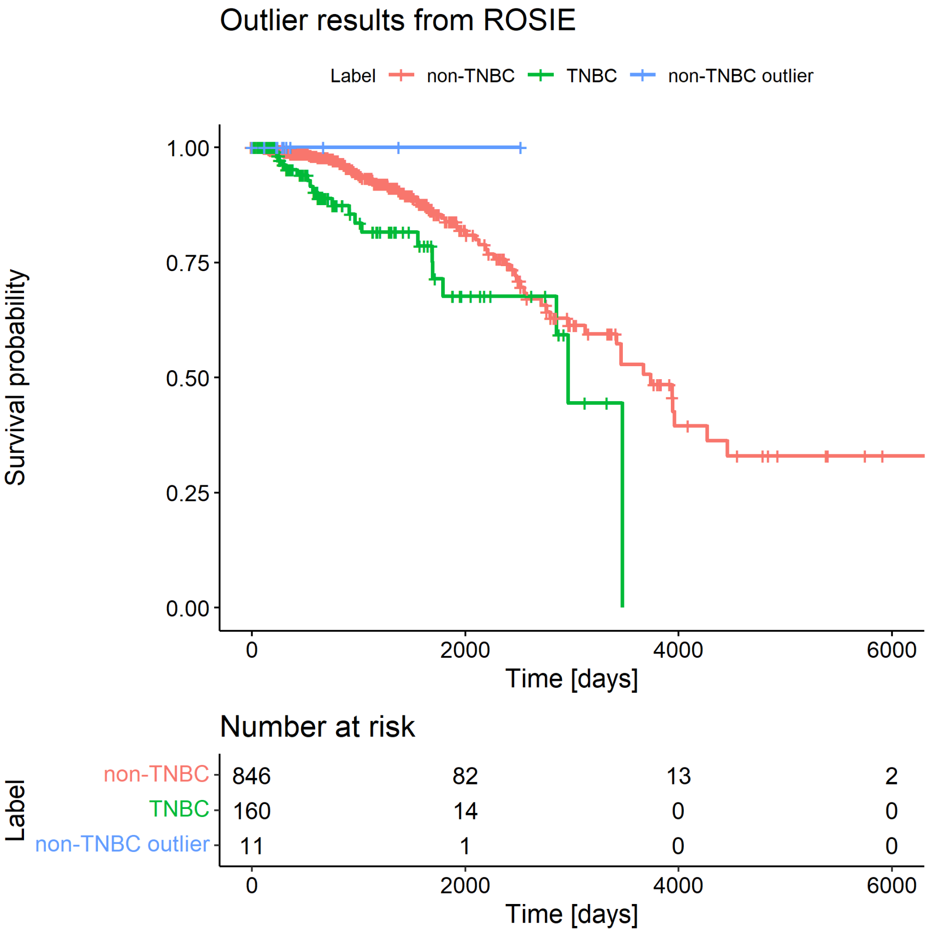

Fig S6: Kaplan-Meier curves and numbers at risk at different time points of TNBC, non-TNBC and outliers

References

1. Hoffmann I, Filzmoser P, Serneels S et al. Sparse and robust pls for binary classification. *Journal of Chemometrics* 2016; 30(4): 153–162. DOI:10.1002/cem.2775. URL https://onlinelibrary.wiley.com/doi/abs/10.1002/cem.2775. https://onlinelibrary.wiley.com/doi/pdf/10.1002/cem.2775.

2. Wold S, Sjöström M and Eriksson L. Pls-regression: a basic tool of chemometrics. *Chemometrics and Intelligent Laboratory Systems* 2001; 58(2): 109 – 130. DOI:https://doi.org/10.1016/S0169-7439(01)00155-1. URL http://www.sciencedirect.com/science/article/pii/S0169743901001551. PLS Methods.

3. Kondo Y, Salibian-Barrera M, Zamar R et al. RSKC: an R package for a robust and sparse k-means clustering algorithm. *Journal of Statistical Software* 2016; 72(5): 1–26.

4. Steinhaus H. Sur la division des corps matériels en parties. *Bull Acad Polon Sci* 1956; 1(804): 801.

5. Witten DM and Tibshirani R. A framework for feature selection in clustering. *Journal of the American Statistical Association* 2010; 105(490): 713–726. DOI:10.1198/jasa.2010.tm09415. URL https://doi.org/10.1198/jasa.2010.tm09415. PMID: 20811510, https://doi.org/10.1198/jasa.2010.tm09415.

6. Cuesta-Albertos JA, Gordaliza A and Matrán C. Trimmed k-means: an attempt to robustify quantizers. *The Annals of Statistics* 1997; 25(2): 553 – 576. DOI:10.1214/aos/1031833664. URL https://doi.org/10.1214/aos/1031833664.

7. Kondo Y. *RSKC: Robust Sparse K-Means*, 2016. URL https://CRAN.R-project.org/package=RSKC. R package version 2.4.2.

8. Kurnaz FS, Hoffmann I and Filzmoser P. Robust and sparse estimation methods for high-dimensional linear and logistic regression. *Chemometrics and Intelligent Laboratory Systems* 2018; 172: 211 – 222. DOI:https://doi.org/10.1016/j.chemolab.2017.11.017. URL http://www.sciencedirect.com/science/article/pii/S0169743917301247.

9. R Core Team *R: A language and environment for statistical computing* R Foundation for Statistical Computing, Vienna, Austria URL https://www.R-project.org/.
